# MRI radiomics-based machine learning for classification of deep-seated lipoma and atypical lipomatous tumor of the extremities

**DOI:** 10.1007/s11547-023-01657-y

**Published:** 2023-06-19

**Authors:** Salvatore Gitto, Matteo Interlenghi, Renato Cuocolo, Christian Salvatore, Vincenzo Giannetta, Julietta Badalyan, Enrico Gallazzi, Maria Silvia Spinelli, Mauro Gallazzi, Francesca Serpi, Carmelo Messina, Domenico Albano, Alessio Annovazzi, Vincenzo Anelli, Jacopo Baldi, Alberto Aliprandi, Elisabetta Armiraglio, Antonina Parafioriti, Primo Andrea Daolio, Alessandro Luzzati, Roberto Biagini, Isabella Castiglioni, Luca Maria Sconfienza

**Affiliations:** 1 IRCCS Istituto Ortopedico Galeazzi, Milan, Italy; 2 Dipartimento di Scienze Biomediche per la Salute, Università degli Studi di Milano, Milan, Italy; 3 DeepTrace Technologies, Milan, Italy; 4 Department of Medicine, Surgery and Dentistry, University of Salerno, Baronissi, Italy; 5 Augmented Reality for Health Monitoring Laboratory (ARHeMLab), Department of Electrical Engineering and Information Technology, University of Naples “Federico II”, Naples, Italy; 6 Department of Science, Technology and Society, University School for Advanced Studies IUSS Pavia, Pavia, Italy; 7 Diagnostic and Interventional Radiology Department, IRCCS Ospedale San Raffaele-Turro, Università Vita-Salute San Raffaele, Milan, Italy; 8 Scuola di Specializzazione in Statistica Sanitaria e Biometria, Università Degli Studi Di Milano, Milan, Italy; 9 UOC Patologia Vertebrale e Scoliosi, ASST Gaetano Pini - CTO, Milan, Italy; 10 UOC Ortopedia Oncologica, ASST Gaetano Pini - CTO, Milan, Italy; 11 UOC Radiodiagnostica, ASST Gaetano Pini - CTO, Milan, Italy; 12 Nuclear Medicine Unit, IRCCS Regina Elena National Cancer Institute, Rome, Italy; 13 Radiology and Diagnostic Imaging Unit, IRCCS Regina Elena National Cancer Institute, Rome, Italy; 14 Oncological Orthopaedics Unit, IRCCS Regina Elena National Cancer Institute, Rome, Italy; 15 Istituti Clinici Zucchi, Monza, Italy; 16 UOC Anatomia Patologica, ASST Gaetano Pini - CTO Milan, Milan, Italy; 17 Department of Physics, Università degli Studi di Milano-Bicocca, Milan, Italy; 18 Institute of Biomedical Imaging and Physiology, Consiglio Nazionale Delle Ricerche, Segrate, Italy

**Keywords:** Artificial intelligence, Lipoma, Liposarcoma, Machine learning, Radiomics, Soft-tissue, Tumor

## Abstract

**Purpose:**

To determine diagnostic performance of MRI radiomics-based machine learning for classification of deep-seated lipoma and atypical lipomatous tumor (ALT) of the extremities.

**Material and methods:**

This retrospective study was performed at three tertiary sarcoma centers and included 150 patients with surgically treated and histology-proven lesions. The training-validation cohort consisted of 114 patients from centers 1 and 2 (n = 64 lipoma, n = 50 ALT). The external test cohort consisted of 36 patients from center 3 (n = 24 lipoma, n = 12 ALT). 3D segmentation was manually performed on T1- and T2-weighted MRI. After extraction and selection of radiomic features, three machine learning classifiers were trained and validated using nested fivefold cross-validation. The best-performing classifier according to previous analysis was evaluated and compared to an experienced musculoskeletal radiologist in the external test cohort.

**Results:**

Eight features passed feature selection and were incorporated into the machine learning models. After training and validation (74% ROC-AUC), the best-performing classifier (Random Forest) showed 92% sensitivity and 33% specificity in the external test cohort with no statistical difference compared to the radiologist (*p* = 0.474).

**Conclusion:**

MRI radiomics-based machine learning may classify deep-seated lipoma and ALT of the extremities with high sensitivity and negative predictive value, thus potentially serving as a non-invasive screening tool to reduce unnecessary referral to tertiary tumor centers.

**Supplementary Information:**

The online version contains supplementary material available at 10.1007/s11547-023-01657-y.

## Introduction

Lipoma and atypical lipomatous tumor (ALT) are the most common soft-tissue neoplasms [1]. Lipomas are benign adipocytic lesions [2]. In the 2020 edition of the World Health Organization classification, the term ALT is reserved for well-differentiated lipomatous lesions located in the extremities, trunk and abdominal wall, where surgery is generally curative. ALTs are categorized as intermediate (locally aggressive) tumors [2]. Lipomatous lesions with the same histology, but located in the retroperitoneum or mediastinum, are defined as well-differentiated liposarcoma (WDLS) and classified within malignant adipocytic tumors based on lower chance of achieving negative surgical margins and higher risk of recurrence and dedifferentiation [2]. The incidence of lipoma and ALT/WDLS is 2/1,000/year and 0.35/100,000/year, respectively [1]. However, in the retroperitoneum, lipomas are very rare and any lipomatous lesion should be considered at least WDLS unless proven otherwise [3]. Conversely, in the extremities, trunk and abdominal wall, lipomas are common [1] and, consequently, the distinction between lipoma and ALT becomes clinically more relevant. Particularly, surgery is the treatment of choice and marginal excision is now the advised option for ALT, whereas lipoma does not require any treatment unless it is symptomatic or there is reason for cosmetic concerns [4].

Because of the different therapeutic options, clinical management depends on our ability to differentiate lipoma from ALT. Biopsy suffers from sampling errors in large ALT and WDLS [5]. Advanced techniques, such as immunohistochemistry and fluorescence in situ hybridization, increase accuracy by identifying MDM2 amplification, which is seen in most ALTs and absent in lipomas [6]. However, although useful in histologically equivocal cases [7], these techniques are time-consuming and expensive [6]. MRI is the imaging method of choice for diagnosis and differentiating lipoma from ALT [8]. However, qualitative MRI evaluation suffers from high interobserver variability [8] and limited accuracy [9]. New imaging-based tools like radiomics have been proposed to characterize lipomatous soft-tissue tumors [10]. Radiomics includes the extraction and analysis of quantitative features from medical images, known as radiomic features, which can be combined with machine learning to create classification models for the diagnosis of interest [11–16].

Lipomatous lesions are categorized as superficial or deep based on their location relative to the fascia overlying the muscles [17]. Deep location is an independent predictor of ALT [8]. In particular, experienced observers with subspecialty training in musculoskeletal radiology or orthopedic oncology have shown to correctly differentiate deep-seated lipoma from ALT/WDLS in 69% of cases based on qualitative MRI assessment [9]. The aim of this study is to determine diagnostic performance of MRI radiomics-based machine learning for classification of deep-seated lipoma and ALT of the extremities.

## Materials and methods

### Ethics

Institutional Review Board approved this multi-center retrospective study and waived the need for informed consent (*protocol name blinded for review*). Patients included in this study granted written permission for anonymized data use for research purposes at the time of the MRI. After matching imaging, pathological, and surgical data, our database was completely anonymized to delete any connection between data and patients’ identity according to the General Data Protection Regulation for Research Hospitals.

### Design and inclusion/exclusion criteria

This retrospective study was conducted at three tertiary sarcoma centers (*center 1, blinded for review; center 2, blinded for review; center 3, blinded for review*). At each center, information was retrieved through medical records from the orthopedic surgery and pathology departments. Patients with ALT or lipoma of the extremities and MRI available at one of the participating centers were considered for inclusion. Inclusion criteria were: (i) deep-seated lipoma or ALT (both intra- and intermuscular lesions, which were located deep to the deep peripheral fascia surrounding muscles [18]) of the extremities that was surgically treated; (ii) definitive pathological diagnosis achieved post-operatively based on both microscopic findings and fluorescence in situ hybridization; (iii) MRI including at least T1- and T2-weighted sequences without fat suppression and fat-suppressed fluid-sensitive sequence in two or more directions performed within 3 months before surgery. Exclusion criteria were: (i) ALT local recurrence; (ii) poor image quality or image artifacts affecting segmentation and machine learning analysis. Overall, 5 patients were excluded at the three centers (n = 1 recurrence; n = 4 poor image quality or artifacts) and 150 patients were finally included in the study.

### Study cohorts

Based on geographical criteria, the training-validation cohort consisted of 114 patients (n = 64 lipoma; n = 50 ALT) from centers 1 and 2 (located in the same city). The external test cohort consisted of 36 patients (n = 24 lipoma; n = 12 ALT) from center 3. Patients’ demographics and data regarding lesion location are detailed in Table 1. In center 1, examinations were performed on one of three 1.5-T MRI systems (Magnetom Espree, Siemens Healthineers, Erlangen, Germany; or Eclipse, Marconi Medical Systems, Cleveland, OH, USA; or Optima MR450w, GE Medical Systems, Milwaukee, WI, USA). In center 2, examinations were performed on one of two 1.5-T MRI systems (Magnetom Avanto, Siemens Healthineers, Erlangen, Germany; or Magnetom Espree, Siemens Healthineers, Erlangen, Germany). In center 3, examinations were performed on a 1.5-T (Optima MR450w, GE Medical Systems, Milwaukee, WI, USA) or 3.0-T (Discovery MR750w, GE Medical Systems, Milwaukee, WI, USA) MRI system. Also, externally obtained MRI scans of patients referred to center 3 were included if the minimal MRI protocol was available. Slice thickness and matrix size ranged from 3 to 6 mm and 256-640 × 220-640, respectively.

**Table 1:** Patients’ age and tumor location in each participating center. Age is presented as median and interquartile (1st–3rd) range

	ALT	LIPOMA	*p*-Value
Center 1			
Age	59 (51–71) years	58 (50–65) years	0.238
Tumor location	Upper extremity: n = 10 Lower extremity: n = 24	Upper extremity: n = 16 Lower extremity: n = 18	0.212
Center 2			
Age	57 (51–70) years	57 (49–65) years	0.772
Tumor location	Upper extremity: n = 2 Lower extremity: n = 14	Upper extremity: n = 15 Lower extremity: n = 15	0.029
Center 3			
Age	60 (53–66) years	55 (48–61) years	0.174
Tumor location	Upper extremity: n = 0 Lower extremity: n = 12	Upper extremity: n = 3 Lower extremity: n = 21	0.522

### Radiomics-based machine learning analysis

Radiomics-based machine learning analysis was performed according to the International Biomarker Standardization Initiative (IBSI) guidelines [19]. The open-source software ITK-SNAP (v3.8) [20] was used for image segmentation. The Trace4Research© radiomic/AI platform (DeepTrace Technologies, www.deeptracetech.com/files/TechnicalSheet__TRACE4.pdf) was used for all subsequent steps of the analysis. In detail, our IBSI-compliant radiomic workflow included several steps as follows.

1. *Segmentation*. A musculoskeletal radiologist performed contour-focused segmentation on T1- and T2-weighted MRI sequences without fat suppression. The axial, as first choice, coronal or sagittal sequences were used based on availability and tumor location. In detail, volumes of interest (VOIs) were manually annotated slice by slice to include the whole tumor. The radiologist knew the study would deal with lipomatous soft-tissue tumors but was blinded to definitive pathological diagnosis.
2. *Image preprocessing*. Preprocessing of image intensities within the segmented VOI included resampling to isotropic voxel spacing (1.5 mm) and intensity discretization using a fixed number of 64 bins.
3. *Extraction of radiomic features*. Features were extracted from the segmented VOI, subdivided into the following families: Morphology, Intensity-based Statistics, Intensity Histogram, Gray-Level Co-occurrence Matrix, Gray-Level Run Length Matrix, Gray-Level Size Zone Matrix, Neighborhood Gray Tone Difference Matrix, Neighboring Gray Level Dependence Matrix. The same features were also extracted from segmented VOI after the application of the following filters: Wavelet, Square, Square-root, Logarithm, Exponential, Gradient, Laplacian of Gaussian. Each filter was applied individually on the original segmented VOI.
4. *Selection of radiomic features*. Selection process provided stable, repeatable, informative, and not redundant features. In detail, features were defined stable with respect to different segmentations and repeatable in test–retest study using ICC (ICC > 0.75) by statistically comparing features obtained by data augmentation strategies, namely randomly manipulating the manual segmentations and rotating the original images and segmentations. Features with low variance (threshold = 0.1) were removed. Highly intercorrelated features were removed by a mutual-information analysis (removing features with mutual information > 0.23).
5. *Training-validation*. In the training-validation cohort, three different models of machine learning classifiers were trained, validated, and internally tested using nested fivefold cross validation. The first model consisted of 10 ensembles of 25 random forest (each random forest composed of 200 decision trees) classifiers combined with Gini index with majority vote rule. The second model consisted of 10 ensembles of 25 support vector machines with linear kernel, combined with principal components analysis and Fisher Discriminant Ratio with majority vote rule. The third model consisted of 10 ensembles of 25 k-nearest neighbor classifiers (5 nearest neighbors for classification) combined with principal components analysis and Fisher Discriminant Ratio with majority vote rule. Oversampling technique for the minority class (ALT) was applied by adaptive synthetic sampling method during model training [21]. The training, validation, and internal testing performances of each model were measured across the folds of cross validation in terms of majority vote and mean ROC-AUC, accuracy, sensitivity, specificity, positive predictive value (PPV) and negative predictive value (NPV) with 95% confidence intervals. For analysis purposes, correctly classified ALT and lipoma were considered as true positive and true negative, respectively. Similarly, incorrectly classified ALT and lipoma were considered as false negative and false positive, respectively. The model showing the best performance in terms of ROC-AUC was chosen as the best classifier.
6. *External testing*. In the external test cohort, the performance of the best classifier (based on step 5 analysis) was finally evaluated using independent data.

### Qualitative image assessment

A musculoskeletal radiologist with 7 years of work experience in a tertiary sarcoma center (*blinded for review*) read all MRI studies from the external test cohort blinded to any information regarding pathology and radiomics-based machine learning analysis. All available MRI sequences were used for qualitative assessment. The following parameters were evaluated to differentiate ALT from lipoma and give the final impression: size, morphology, thick septations and non-fatty components showing incomplete fat suppression [8].

### Statistical analysis

Statistical analysis was performed using the Trace4Research platform. The medians and 95% confidence intervals of the selected radiomic predictors were calculated in the two classes “ALT” and “lipoma”. Mann–Whitney U test was performed to explore statistical differences between these two classes. To account for multiple comparisons, p-values were adjusted using the Bonferroni-Holm method. Mann–Whitney U and Chi-square tests were used to evaluate differences in age and tumor location between ALT and lipoma, respectively. In the external test cohort, machine learning performance was compared to qualitative MRI assessment using McNemar’s test. A two-sided *p*-value < 0.05 indicated statistical significance. A radiologist with experience in radiomics (blinded for review) assessed Radiomics Quality Score in the attempt to estimate the methodological rigor of our study, as suggested by Lambin et al. [22].

## Results

**Fig. 1:**
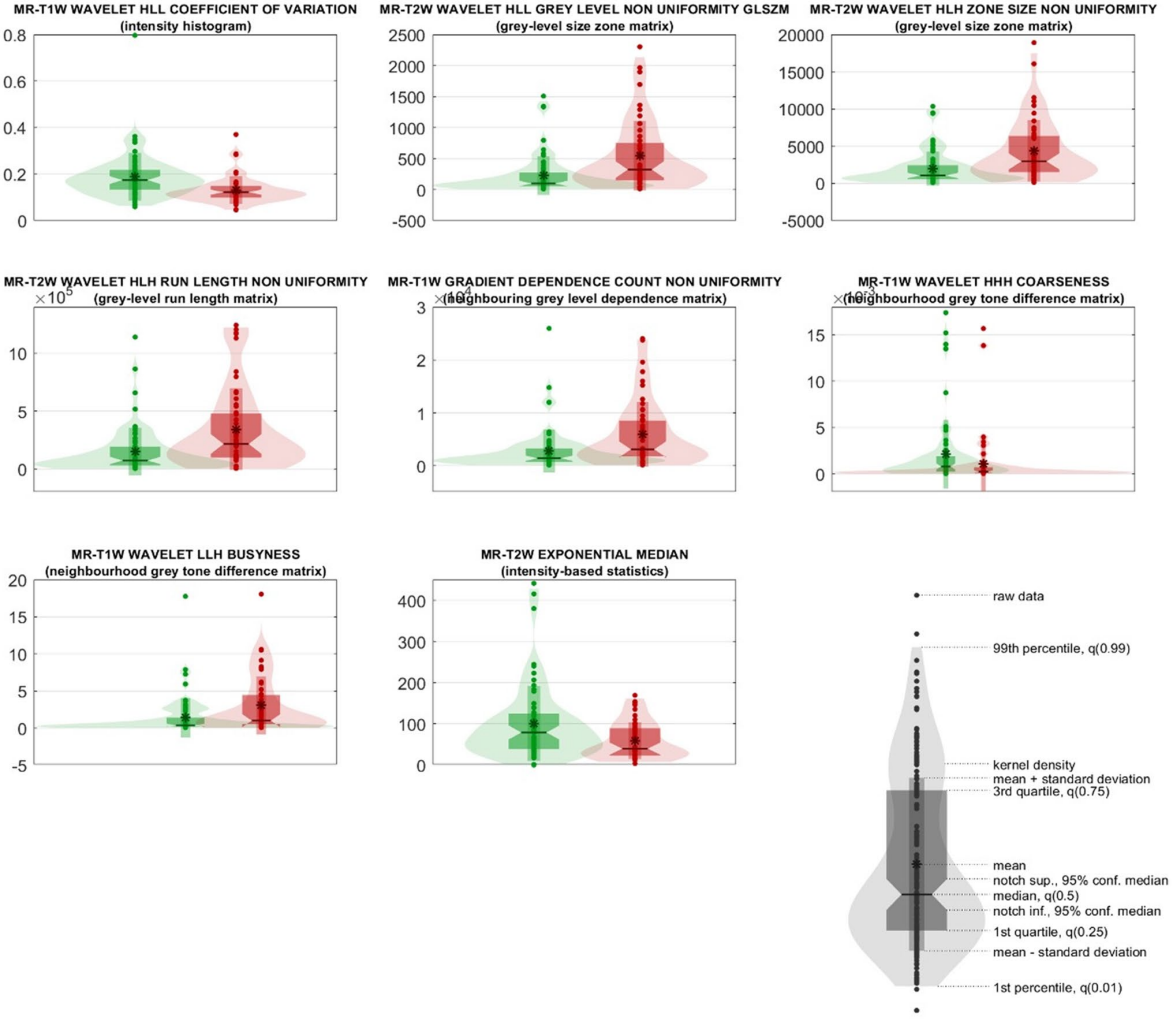
Violin and box plots of the radiomic predictors ranked from 1 to 8. Violin and box plots of “ALT” and “lipoma” classes are reported in red and green, respectively

No age difference was found between ALT and lipoma in any of the participating centers (*p* > 0.174). Tumor location was not different between the two classes in centers 1 and 3 (*p* > 0.212), whereas lower extremity location was significantly associated (*p* = 0.029) with ALT in center 2 (Table 1). After extraction of 3,380 IBSI-compliant radiomic features belonging to the families previously described, 2052 resulted stable and repeatable; of these, 1724 resulted having variance above 0.10. Finally, eight features resulted poorly intercorrelated (mutual information below 0.23), passing feature selection, and were incorporated into the machine learning models. The selected features are detailed in Table 2, along with their median values and confidence intervals in the two classes “ALT” and “lipoma”. Their distribution is shown in violin and box plots in Fig. 1.

**Table 2:** Selected radiomic features reported in descending order according to their statistical significance and relevance in the ensemble of random forest classifiers

#	Feature family	Feature nomenclature	Median in the ALT class [95% CI]	Median in the lipoma class [95% CI]	Uncorrected p-value	Corrected p-value
1	Intensity Histogram	MR-T1W_wavelet_HLL_coefficient Of Variation	0.12 [0.11–0.14]	0.17 [0.16–0.19]	< 0.005	< 0.005
2	Grey-Level Size Zone Matrix	MR-T2W_wavelet_HLL_grey Level Non Uniformity Glszm	322.39 [180.42–464.36]	98.49 [55.31–141.68]	< 0.005	< 0.005
3	Grey-Level Size Zone Matrix	MR-T2W_wavelet_HLH_zone Size Non Uniformity	2947.81 [1849.57–4046.05]	1052.93 [685.96–1419.91]	< 0.005	< 0.005
4	Grey-Level Run Length Matrix	MR-T2W_wavelet_HLH_Run Length Non Uniformity	2.18e + 05 [1.31e + 05–3.04e + 05]	7.51e + 04 [4.35e + 04–1.07e + 05]	< 0.005	< 0.005
5	Neighbouring Grey Level Dependence Matrix	MR-T1W_gradient_dependence Count Non Uniformity	3113.94 [1612.15–4615.74]	1440.7 [948.13–1933.27]	< 0.005	< 0.005
6	Neighbourhood Grey Tone Difference Matrix	MR-T1W_wavelet_HHH_coarseness	2.19e-04 [7.40e-05–3.64e-04]	7.95e-04 [4.67e-04–1.12e-03]	< 0.005	< 0.005
7	Neighbourhood Grey Tone Difference Matrix	MR-T1W_wavelet_LLH_busyness	0.99 [9.07e-02–1.89]	0.34 [0.1–0.58]	< 0.005	< 0.05
8	Intensity-Based Statistics	MR-T2W_exponential_median	39.79 [24.71–54.86]	79.08 [62.1–96.06]	< 0.005	< 0.05

**Fig. 2:**
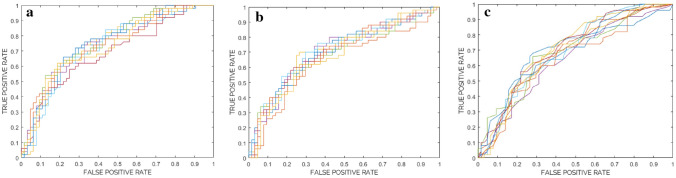
ROC curves for the models consisting of 10 ensembles of Random Forest (**a**), Support Vector Machine (**b**) and k nearest neighbors (**c**) classifiers from internal testing

Table 3 details the performance of each model assessed in the training-validation cohort. The ROC curves for each model consisting of 10 ensembles of Random Forest, Support Vector Machine and k nearest neighbors classifiers (from internal testing) are plotted in Fig. 2a–c, respectively. Specifically, the best classifier (Random Forest—majority vote with 38.9% threshold) showed 74% ROC-AUC and was chosen for further analysis.

**Table 3:** Models of 10 ensembles of random forest, support vector machine and k nearest neighbors classifiers. Classification performance is reported for training, validation, and internal testing in terms of ROC-AUC, accuracy, sensitivity, specificity, PPV, NPV, including 95% confidence intervals (CI), and statistical significance with respect to chance/random classification

	Training	Validation	Internal testing (mean)	Internal testing (majority vote—38.9% threshold)
Random forest				
ROC-AUC (%) [95% CI]	100* [99–100]	73** [72–74]	74** [72–75]	74
Accuracy (%) [95% CI]	100* [99–100]	67** [67–68]	69** [68–70]	68
Sensitivity (%) [95% CI]	100* [99–100]	62** [60–63]	64** [62–66]	78
Specificity (%) [95% CI]	100* [99–100]	72** [71–73]	73** [71–74]	61
PPV (%) [95% CI]	100* [99–100]	64** [63–65]	65** [63–66]	61
NPV (%) [95% CI]	100* [99–100]	71** [71–72]	72** [71–73]	78
Support vector machine				
ROC-AUC (%) [95% CI]	75** [74–76]	74** [73–74]	69** [68–71]	71
Accuracy (%) [95% CI]	68** [67–69]	67** [66–68]	65** [64–66]	68
Sensitivity (%) [95% CI]	47** [45–49]	46** [44–48]	42** [39–46]	68
Specificity (%) [95% CI]	85** [84–85]	84** [83–84]	83** [81–84]	69
PPV (%) [95% CI]	70** [69–71]	71** [70–73]	66** [63–68]	63
NPV (%) [95% CI]	67** [66–68]	67** [66–68]	65** [63–66]	73
K nearest neighbors				
ROC-AUC (%) [95% CI]	83** [82–83]	69** [67–70]	68** [67–70]	70
Accuracy (%) [95% CI]	76** [75–76]	65** [64–67]	65** [63–67]	67
Sensitivity (%) [95% CI]	70** [68–71]	57** [55–59]	55** [51–59]	80
Specificity (%) [95% CI]	80** [79–81]	72** [70–73]	73** [71–74]	56
PPV (%) [95% CI]	74** [73–74]	62** [61–64]	61** [59–63]	59
NPV (%) [95% CI]	78** [77–78]	69** [67–70]	67** [65–69]	78

*p-value < 0.05/**p-value < 0.005

**Fig. 3:**
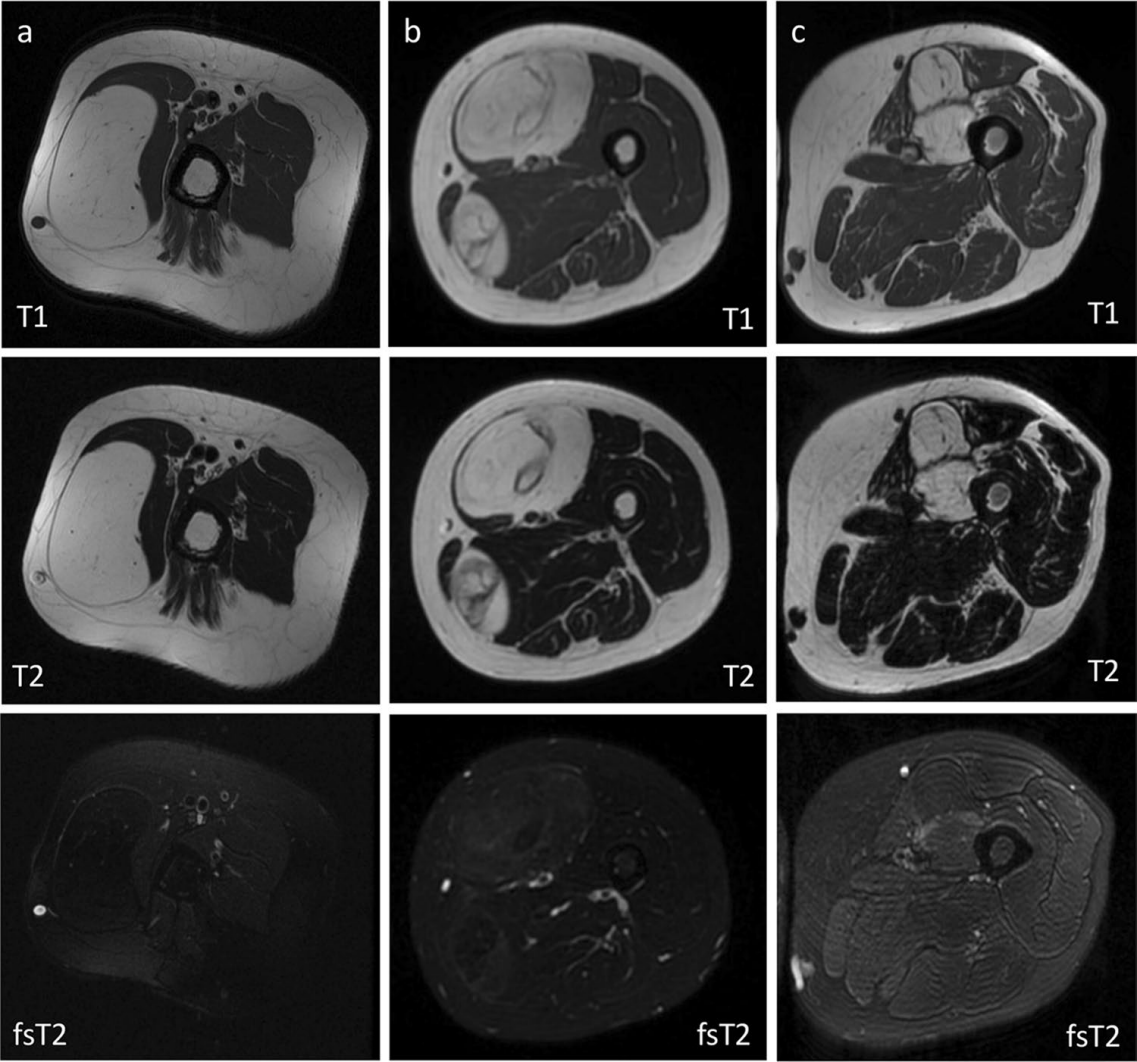
True negative (**a**, correctly classified lipoma), false positive (**b**, lipoma misdiagnosed as ALT) and true positive (**c**, correctly classified ALT) according to both radiomics-based machine learning and qualitative assessment performed by the radiologist. In (**a**), correctly classified lipoma shows homogeneous signal with complete fat suppression. Intralesional septations are seen in correctly classified ALT (**c**) but also lipoma misdiagnosed as ALT (**b**). Fat-suppressed T2-weighted sequences were used only for qualitative assessment performed by the radiologist. Radiomics-based machine learning analysis included T1- and T2-weighted sequences without fat suppression

Table 4 details the performance of the best model (Random Forest—majority vote with 38.9% threshold) in the external test cohort. In particular, the model showed 92% sensitivity and 33% specificity in differentiating ALT from lipoma. The radiologist had 88% sensitivity and 54% specificity with no statistical difference compared to machine learning (*p* = 0.474), as reported in Table 4. Figure 3 shows examples of true negative (correctly classified lipoma), false positive (lipoma misdiagnosed as ALT) and true positive (correctly classified ALT) according to both radiomics-based machine learning and qualitative assessment performed by the radiologist. Our Radiomics Quality Score was 39% (Supplementary material).

**Table 4:** Model of 10 ensembles of random forest classifiers. Classification performance is reported for external testing in terms of accuracy, sensitivity, specificity, PPV and NPV

External testing	Random forest (majority vote—38.9% threshold)	Radiologist
Accuracy (%)	53 (19/36)	64 (23/36)
Sensitivity (%)	92 (11/12)	88 (10/12)
Specificity (%)	33 (8/24)	54 (13/24)
PPV (%)	41 (11/27)	48 (10/21)
NPV (%)	89 (8/9)	87 (13/15)

p-value = 0.474 (machine learning vs. radiologist)

## Discussion

Our study addressed the issue of differentiating deep-seated lipoma from ALT of the extremities using MRI radiomics-based machine learning models, which were trained, validated, and tested against independent data from an external dataset. Our main finding is that our best model (10 ensembles of Random Forest classifiers) showed very high sensitivity and NPV in the external test cohort, which respectively amounted to 92% and 89%, with no difference compared to a dedicated musculoskeletal radiologist (*p* = 0.474). Thus, if lesions were classified as lipoma (negative group) using machine learning, further work-up could be spared and related costs could be saved. This could be especially useful in peripheral hospitals where personnel have no experience and expertise in soft-tissue tumors, thus avoiding patients’ worry and referral to tertiary sarcoma centers when unneeded. Our model’s performance including higher sensitivity and NPV than specificity and PPV is also in line with visual MRI reading performed by experts [9] and highlights the difficulty of differentiating deep-seated lipoma from ALT based on both qualitative imaging assessment and radiomics-based machine learning analysis.

Previous studies focused on MRI radiomics of lipomatous soft-tissue tumors, either alone [23] or combined with machine learning [10]. In particular, Thornhill et al. [24] and Malinauskaite et al. [25] performed radiomic analyses in relatively small groups of patients (n = 44 and n = 38, respectively) to distinguish between lipoma and liposarcoma. However, in addition to ALT/WDLS, the latter group included dedifferentiated and myxoid liposarcomas, which are more easily differentiated from lipoma on qualitative MRI analysis performed by radiologists [17]. Other authors only included lipoma and ALT/WDLS, which are the most challenging lipomatous soft-tissue lesions to discriminate between, as we did in our current work. These studies performed radiomic analyses based on either non-enhanced T1- and T2-weighted [26–28] or contrast-enhanced T1-weighted [29] MRI sequences, including population ranging from 65 to 122 subjects and achieving AUCs of 0.83 or higher. Nonetheless, model performance was not validated using independent data from different centers in all these studies.

In a single-center study, Cay et al. [26] showed better performance than previous works when a single type of MRI scanner and consistent presets were used for radiomics-based machine learning analysis. Hence, the authors concluded that accuracy of radiomic approaches could be improved using standardized hardware and imaging protocols [26]. However, a main challenge of radiomics is the absence of standardized image acquisition protocols between different centers [30], thus advocating the need for model validation. A clinical validation against independent datasets is essential to evaluate model generalizability and promote its application to real-world settings [31]. An independent clinical validation on an external dataset was recently provided in the study by Fradet et al. [32], which investigated contrast-enhanced MRI radiomics-based machine learning for lipoma/ALT differentiation. This study included a heterogenous group of 145 patients with images collected at many centers using non-uniform protocols and centralized at two institutions, which constituted the training and external test cohorts, respectively. In the external test cohort, the authors reported a sharp decrease in model performance with AUCs ranging from 0.47 to 0.71, although some improvement was obtained through statistical harmonization using batch effect correction [32]. High sensitivity and limited specificity were reported for the best classifier [32], as we also observed in our study. Based on their and our findings, we believe that high heterogeneity in the images of ALT and lipoma obtained from various body regions and different MRI scanners and protocols makes the task of generalization difficult. Fradet et al. also evaluated deep learning approaches, which were outperformed by radiomics-based classical machine learning [32]. However, the use of deep learning for lipoma/ALT differentiation is at early stages, with another study reporting its superior accuracy compared to classical machine learning [33] and thus warranting future investigation.

Some limitations of this study need to be addressed. First, the study design was retrospective. Although prospective studies provide the highest level of evidence supporting the clinical validity and usefulness of radiomics [22], a retrospective design allowed including relatively large numbers of patients with imaging data already available. Second, a selection bias existed in our study, as lipomas were included only if seen at tertiary sarcoma centers (any of the participating centers) and surgically treated. Lipomas are usually neither referred to sarcoma centers nor operated if they are small or show no suspicious imaging features. However, this probably made the dataset even more challenging and relevant, as only the most complex cases were included. Third, lipomas were over-represented compared to ALTs in our population of study. However, this reflects the incidence of lipoma and ALT [1], and class balancing was performed to artificially oversample the minority class in the training cohort [21]. Fourth, the retrospective design accounted for the exclusion of contrast-enhanced MRI, as contrast is not routinely administered for lipoma/ALT at two of the participating centers. This is in line with studies suggesting that the value of contrast administration may be limited in lipoma and ALT [8]. Additionally, other authors recently evaluated contrast-enhanced MRI radiomics for lipoma/ALT differentiation and validated their machine learning model using an independent external dataset [32], with similar findings compared to our approach based on non-contrast MRI only. Finally, our radiomics quality score was 39%. This is in line with the mean values reported in a recent systematic review of the radiomics quality score applications [34], but highlights that methodological quality can still be improved in the future.

In conclusion, MRI radiomics-based machine learning may differentiate deep-seated lipoma from ALT of the extremities with high sensitivity and NPV. Although specificity is still limited, our model’s performance is in line with visual MRI reading performed by experts, as reported in literature [9] and also observed in our study. Hence, our approach may serve as a screening tool in hospitals where radiologists have no experience and expertise in soft-tissue tumors, thus avoiding unnecessary referral to tertiary sarcoma centers and invasive procedures such as biopsy. Additionally, larger multi-center studies are needed to address the issue of MRI scanner/protocol variability and possibly highlight the need for machine learning model re-training/validation in different institutions.

## Supplementary Information

Below is the link to the electronic supplementary material.

Supplementary file1 (PDF 51 kb)

Supplementary file2 (TIF 5090 kb)

Supplementary file3 (TIF 19597 kb)

Supplementary file4 (TIF 11072 kb)
