# Supplementary material for: MRI radiomics-based machine learning for classification of deep-seated lipoma and atypical lipomatous tumor of the extremities
Source: Radiol Med. 2023 Jun 19;128(8):989–98. doi: 10.1007/s11547-023-01657-y (PMC10338387; doi:10.1007/s11547-023-01657-y)
Supplement: Supplementary file 1 — Supplementary file1 (PDF 51 kb) [file 11547_2023_1657_MOESM1_ESM.pdf]

MRI radiomics-based machine learning for classification of deep-seated lipoma and atypical lipomatous tumor of the extremities

	Radiomics Quality Score
**Item 1**	1
**Item 2**	1
**Item 3**	0
**Item 4**	0
**Item 5**	3
**Item 6**	0
**Item 7**	0
**Item 8**	0
**Item 9**	2
**Item 10**	0
**Item 11**	0
**Item 12**	3
**Item 13**	2
**Item 14**	2
**Item 15**	0
**Item 16**	0
**total**	14
**total (%)**	38,89

Reference: Lambin et al. Radiomics: the bridge between medical imaging and personalized medicine.
Nat Rev Clin Oncol 2017;14:749-762. doi: 10.1038/nrclinonc.2017.141
