# Supplementary figures and images for: MRI radiomics-based machine learning for classification of deep-seated lipoma and atypical lipomatous tumor of the extremities
Source: Radiol Med. 2023 Jun 19;128(8):989–98. doi: 10.1007/s11547-023-01657-y (PMC10338387; doi:10.1007/s11547-023-01657-y)

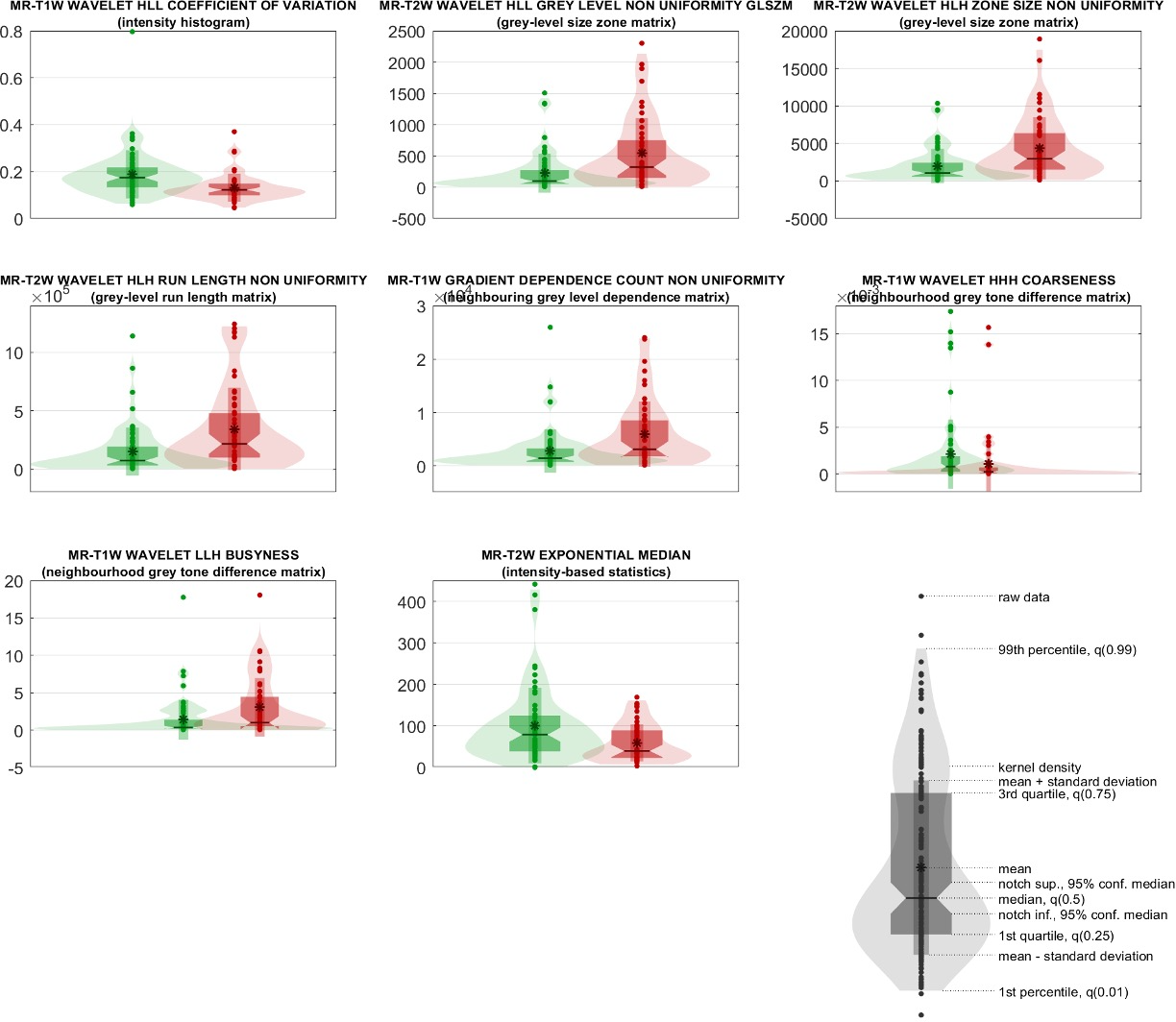

Supplement: Supplementary file 2 — Supplementary file2 (TIF 5090 kb) [file 11547_2023_1657_MOESM2_ESM.tif]

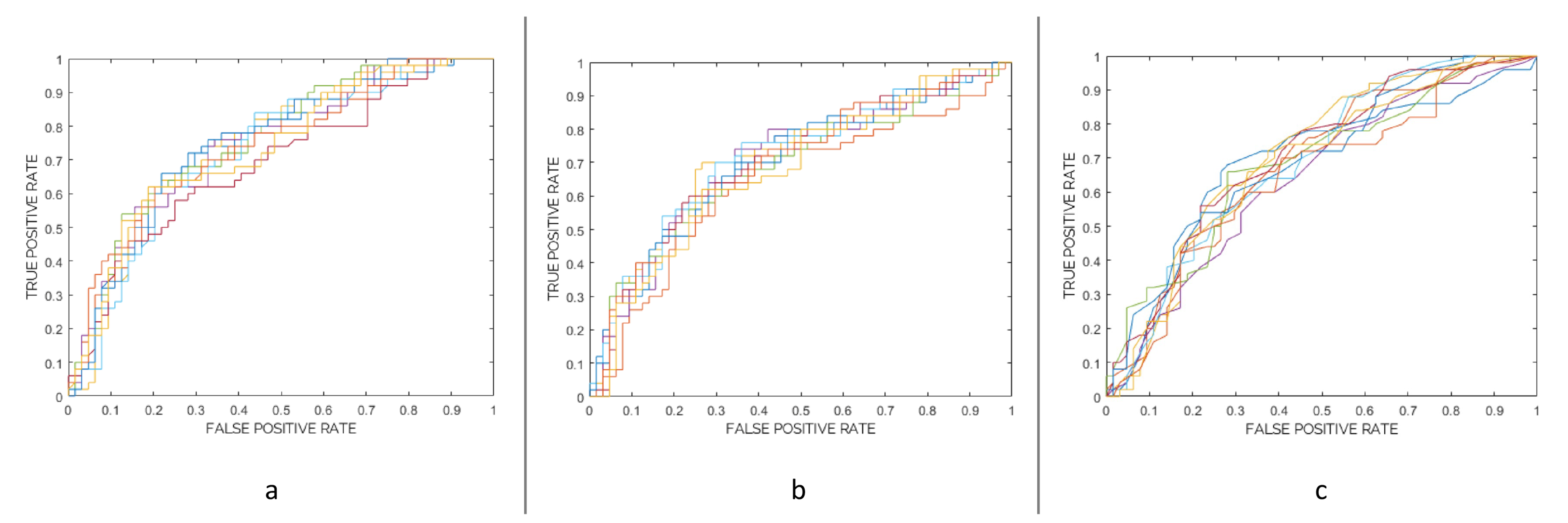

Supplement: Supplementary file 3 — Supplementary file3 (TIF 19597 kb) [file 11547_2023_1657_MOESM3_ESM.tif]

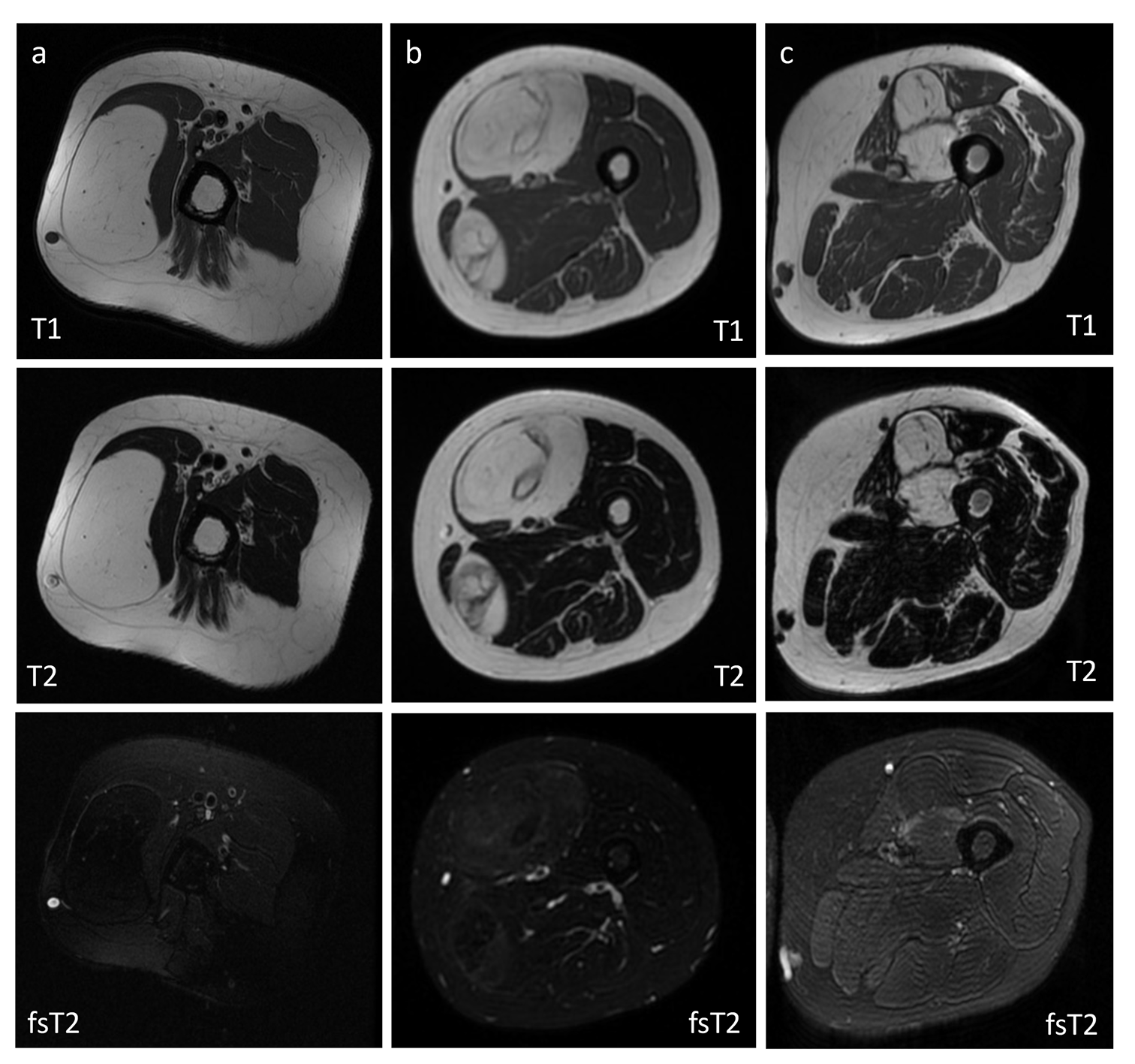

Supplement: Supplementary file 4 — Supplementary file4 (TIF 11072 kb) [file 11547_2023_1657_MOESM4_ESM.tif]
